# Gut microbiota-motility interregulation: insights from *in vivo, ex vivo* and *in silico* studies

**DOI:** 10.1080/19490976.2021.1997296

**Published:** 2022-01-03

**Authors:** Barbora Waclawiková, Agnese Codutti, Karen Alim, Sahar El Aidy

**Affiliations:** a Host-Microbe Interactions, Groningen Biomolecular Sciences and Biotechnology Institute (GBB), University of Groningen, Groningen, The Netherlands; b Max Planck Institute for Dynamics and Self-Organization, Göttingen, Germany; c Physics Department and Center for Protein Assemblies (CPA), Technische Universität München, Garching, Germany

**Keywords:** Microbiota, gut motility, bacterial metabolites, computational modeling

## Abstract

The human gastrointestinal tract is home to trillions of microbes. Gut microbial communities have a significant regulatory role in the intestinal physiology, such as gut motility. Microbial effect on gut motility is often evoked by bioactive molecules from various sources, including microbial break down of carbohydrates, fibers or proteins. In turn, gut motility regulates the colonization within the microbial ecosystem. However, the underlying mechanisms of such regulation remain obscure. Deciphering the inter-regulatory mechanisms of the microbiota and bowel function is crucial for the prevention and treatment of gut dysmotility, a comorbidity associated with many diseases. In this review, we present an overview of the current knowledge on the impact of gut microbiota and its products on bowel motility. We discuss the currently available techniques employed to assess the changes in the intestinal motility. Further, we highlight the open challenges, and incorporate biophysical elements of microbes-motility interplay, in an attempt to lay the foundation for describing long-term impacts of microbial metabolite-induced changes in gut motility.

## Introduction

Understanding gut motility is fundamental when addressing functional gastrointestinal disorders, including inflammatory bowel disease and constipation. A large-scale multinational study revealed that more than 40% of the population worldwide suffer from functional gastrointestinal disorders, which, consequently, have a negative impact on the quality of life and health care use^1^. Gastrointestinal motility is a key physiological parameter that governs digestion and absorption of nutrients, and is regulated by various factors, such as the enteric nervous system, immune system, gut hormones, as well as gut microbes.^2–4^ Nonetheless, the mechanisms that govern the complex and dynamic interrelationships between these factors remain unclear. To date, reports on the mechanisms underlying gut motility typically comes from three types of research methodologies; 1) *in vivo* investigations, including human or animal studies,^4–7^ 2) *in vitro/ex vivo* experiments, which involve cell/organ culture, and organ bath studies,^4,5,8^ and 3) *in silico* models, which employ simulations and computational methods in an attempt to cover different aspects of interactions between motility, flow, transport, bacteria, and their metabolites. This review summarizes current literature available on the impact of the microbiota on gut motility and describes how *in silico* studies can be designed to complement the existing methods to allow deciphering the mechanisms underlying the microbiota-gut motility interplay.

## Regulation of the gastrointestinal motility

**Figure 1.**
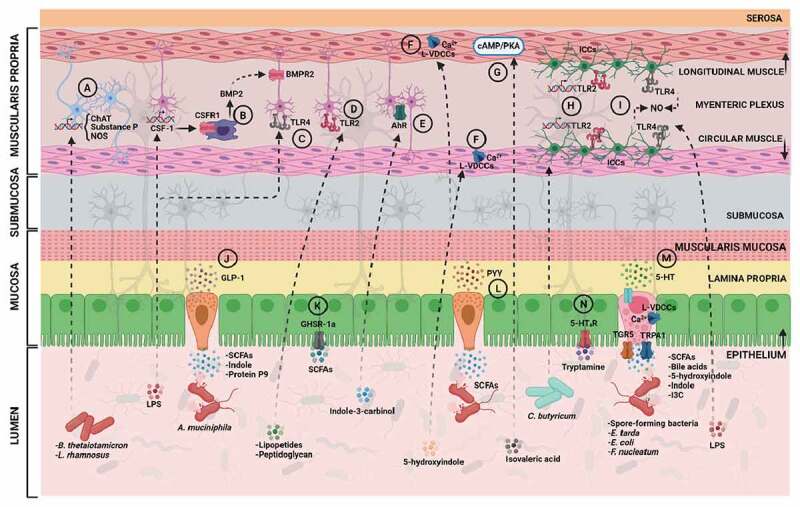
**Gut microbiota, its metabolites and impact on gut motility**. Potential routes by which the gut microbiota can influence intestinal motility via different mechanisms and pathways located in the gut epithelium, circular or longitudinal muscles, or myenteric plexus. Abbreviations: CSF-1, colony stimulatory factor 1; CSFR1, colony stimulatory factor 1 receptor; BMP2, bone morphogenetic protein 2; BMPR2, bone morphogenetic protein 2 receptor; AhR, aryl hydrocarbon receptor; ChAT, choline acetyltransferase; NOS, nitric oxide synthase; L-VDCCs, L-type voltage-dependent calcium channels; TLR2, toll-like receptor 2; ICCs, interstitial cells of Cajal; TLR4, toll-like receptor 4; NO, nitric oxide; 5-HT_4_R, serotonin receptor 4; SCFAs, short-chain fatty acids; GLP-1, glucagon-like peptide 1; TGR5, G-protein coupled bile acid receptor; TRPA1, transient receptor potential cation channel 1; 5-HT, serotonin; PYY, peptide YY; LPS, lipopolysaccharides. (Created with bioRender.com).

Gastrointestinal motility refers to the digestive motor function and the transit of ingested material throughout the gastrointestinal tract.^9^ It involves the coordination of smooth muscle and nerve function to mix, triturate, and propel products of digestion. Digestion and motility are facilitated by the collaborative work of different parts of the digestive tract; the esophagus, stomach, small, and large intestine. Gut motility is regulated via a multitude of regulatory elements, including enteric neurons, smooth muscle cells, interstitial cells, hormones, and particular stimuli, such as gut bacteria and their metabolites.^2–4,10,11^ Additionally, there are various timescales that govern the regulation of the above-mentioned elements and gut motility. For example, changes in the innervation of the gastrointestinal tract, or signaling in the enteric nervous system and neurogenesis, occur in a timescale ranging from several minutes^12^ to several weeks,^13^ respectively. Induction of gene expression in response to receptor activation, however, happens in a timescale of seconds to microseconds.^12^ The unique architecture of the human gastrointestinal tract facilitates all the components necessary for the precise functioning of gut motility^14^ (Figure 1). For example, the gastrointestinal wall is composed of several layers protruded by enteric neurons, those are the mucosa (epithelium, lamina propria, and muscularis mucosa), the submucosa (submucosal plexus), the muscularis propria (circular layer of the smooth muscle, myenteric plexus, and longitudinal layer of the smooth muscle), and the serosa.^14^ The movement of the muscles underlying the propulsion of content is coordinated by the myenteric plexus, while the submucosal plexus is broadly involved in secretion and absorption.^14^ Unraveling the fundamental mechanisms underlying the regulation of gastrointestinal motility is required to understand the causes of bowel dysfunction.

Why is the figure not in color?

The gastrointestinal tract is the only organ that has evolved with its independent nervous system, known as the enteric nervous system. The enteric nervous system was uncovered in 1755 by Albert Von Haller, who stated that “*the intestines in this state after being deprived from all communication with the brain, preserve their peristaltic motion*”.^15^ Nonetheless, although the enteric nervous system can function independent of the central nervous system, under physiological conditions, the bowel motility is also influenced by the central nervous system.^16^ In humans, the enteric nervous system comprises 200–600 million neurons, the great majority of which are found in the myenteric and submucosal plexus.^17^ Recently, profiling of the enteric nervous system in the colon of adult mice enabled the identification of 21 neuronal types.^18^ Among those 21 colonic neuronal types are: (1) sensory neurons (4 subsets), also referred to as intrinsic primary afferent neurons (IPANs), which sense and respond to chemical and mechanical stimuli in the intestine; (2) interneurons (3 subsets), which relay signals between neurons; (3) secretomotor/vasodilator neurons (2 subsets), which trigger secretions and fluid movement in other cell types; (4) excitatory motor neurons (5 subsets) and (5) inhibitory motor neurons (7 subsets), which together coordinate muscle contraction and relaxation and innervate longitudinal and circular muscles in the gastrointestinal tract.^18^ Interestingly, the neuronal types differ between various regions of the gastrointestinal tract as well as between various species.^18^ For example, mouse ileum contains 12 neuronal types, while human colon contains 14 neuronal types.^18^

The primary neurotransmitters of the excitatory neurons are the neuropeptides, acetylcholine and tachykinins, acetylcholine responses are mediated by the muscarinic receptors (M_2_ and M_3_), tachykinins (substance P and neurokinin A) bind to NK1 and NK2 receptors and activate pathways similar to those that involve acetylcholine.^19–28^ The inhibitory neurons constitute multiple transmitters, including nitric oxide, vasoactive intestinal peptide (VIP), and ATP-like transmitters.^17,29^ The nitric oxide is considered as the predominant inhibitory neurotransmitter, and deficits in transmission are observed in its absence as demonstrated in models where nitric oxide synthase is knocked out.^2,30^ The role of the enteric nervous system in the regulation of gut motility has been extensively reviewed elsewhere.^3^ Here, we will focus on the less well-studied role of the gut microbiota and its metabolites as a key regulator of the gut motility.

### Gut motility is highly dependent on the microbiota

Studies investigating the role of microbiota in regulating the gut motility started decades ago, when researchers observed up to 10 times larger cecum in germ-free rats compared to conventional rats, in addition to delayed gastric emptying, suggesting a strong role for the microbiota in the development of a normal gut motility.^31–34^ Since then, germ-free animals have been used in many studies to investigate the influence of the microbiota on gut motility. For example, Husebye et al. demonstrated that germ-free rats displayed a significant delay in the intestinal transit and in the contractility of the small intestine compared to their conventional counterparts, and this delay was partially reversed after colonization with *Lactobacillus acidophilus* together with *Bifidobacterium bifidum*.^35^ Moreover, germ-free mice were found to have a reduced number of colonic neurons in the enteric nervous system compared to their conventional controls.^36^ Analogously, germ-free mice were reported to have lower excitability in the myenteric IPANs, which was normalized when these mice were conventionalized.^37^ Mice treated with antibiotics had significantly lower defecation frequency and slower total gut transit time.^38,39^ Ge et al. also showed that the contractility of the proximal colonic longitudinal muscles was inhibited in the antibiotics-treated mice compared to the untreated controls.^38^ Altogether, these studies demonstrate a significant role of gut microbes in controlling the gut motility.

### Bacterial modulation of the gut motility via the enteric neurons and immune system

Emerging evidence is pointing to the impact of the gut microbiota on the complex signaling of the enteric nervous system.^36,39–41^ For example, the abundant human resident gut bacterium, *Bacteroides thetaiotamicron*, was shown to be critical for the enteric nervous system innervation in the colon.^42^ In that study, colonization of germ-free mice with *Bacteroides thetaiotamicron* restored the expression of excitatory and inhibitory motor neuron signaling enzymes, such as choline acetyltransferase (responsible for synthesis of acetylcholine), substance P, and nitric oxide synthase^42^ (Figure 1(A)). Moreover, when the authors used the pan-neuronal marker, PGP9.5 to investigate the changes in the neuronal innervation between germ-free and specific pathogen-free mice, they observed a reduction in the overall neuronal innervation in both the colonic mucosa and muscle layers (including the myenteric plexus) in germ-free mice.^42^ Similarly, when *Lactobacillus rhamnosus* GG was administered to mice, the expression of choline acetyltransferase was elevated^43^ (Figure 1(A)).

Since the enteric nervous system and gut microbiota reside in close proximity to the intestinal mucosal immune system, it is not surprising that the latter plays a role in modulating the bacterial influence on gut motility. Indeed, Muller et al. observed a role of a subtype of macrophages that reside in the muscularis mucosa, namely muscularis macrophages, in regulating the peristaltic activity of the colon in mice.^40^ Muscularis macrophages could change the pattern of colonic contractility by releasing a growth factor, BMP2, which activates BMP receptor expressed on the enteric neurons (Figure 1(B)). In response, the enteric neurons release a growth factor CSF-1 required for macrophages development^40^ (Figure 1(B)). Muller et al. showed that this immune-neuronal crosstalk is induced by intestinal microbial stimuli, in particular, the microbial cellular component, lipopolysaccharide (LPS), which regulates BMP2 and CSF-1 expression and may, in turn, alter intestinal motility^40^ (Figure 1(B)).

Similarly, two subtypes of the Toll-like receptors (TLRs), TLR2 and TLR4, were recently linked to the regulation of murine intestinal enteric neurons, and gut motility^36,44^ (Figures 1C**, 1D**). Yarandi et al. observed that the inhibition of endogenous signaling of TLR2 (which recognizes lipopeptides and peptidoglycan^45^), *in vivo*, resulted in inhibition of neurogenesis in healthy mice, and, in turn, in significant dysmotility and loss of colonic myenteric neurons.^44^ Anitha et al. reported that the lack of TLR4 signaling (which recognizes LPS) led to delayed gastrointestinal motility in mice as demonstrated by reduced fecal pellet frequency and delayed intestinal transit.^36^ Furthermore, the aryl hydrocarbon receptor (AhR), which is directly activated by gut microbial metabolites (discussed in more details in section *Tryptophan metabolites*),^46^ and is considered a key component of the immune response at barrier sites,^47^ has been recently shown to be involved in the regulation of murine total intestinal transit time in response to microbiota-derived metabolites^39^(Figure 1(E)). Indeed, microbiota-induced expression of AhR in the colonic neurons were shown to be involved in the induction of response of these neurons to the luminal environment, specifically to the microbiota-derived AhR agonist indole-3-carbinol.^39^ Overall, these studies provide invaluable information on the immunological control of intestinal motility and on the basis for understanding the pathophysiology of gastrointestinal motility disorders.

Not only gut commensals but also several pathogens such as *Vibrio cholerae* and *Salmonella typhimurium*, can influence gastrointestinal motility via distinct mechanisms.^48,49^ For example, *V. cholerae* use the syringe-like type VI secretion system to induce intestinal movements in zebrafish, which led to expulsion of the resident microbiota by the host.^48^ *S. typhimurium*-derived enterotoxin caused dramatic changes in intestinal myoelectric activity in white albino rabbits and substantial fluid production after injection of *E. coli* lysates containing the cloned *S. typhimurium* enterotoxin to the ileal loops.^49^

### Bacterial interaction with the gastrointestinal smooth muscles

Throughout the gastrointestinal tract, smooth muscles are organized into two layers of circularly- and longitudinally-oriented muscle bundles to form electrical and mechanical junctions between cells that facilitate coordination of contractions^2^ (Figure 1). Excitation and contraction of gut muscles are regulated by membrane depolarization (influx of positively charged ions inside the cell), which activates voltage-dependent Ca^2+^ channels and triggers elevated levels of intracellular Ca.^2+50–54^ Gastrointestinal smooth muscles express mostly L-type voltage-dependent Ca^2+^ channels, encoded by the *CACNA1C* gene.^55^ However the T-type Ca^2+^ channels also contribute to the Ca^2+^ influx in these muscles.^56^ Recently, we showed how a novel gut bacteria-derived metabolite, 5-hydroxyindole, affects the total gut transit time in rats via modulating L-type voltage-dependent Ca^2+^ channels located on the colonic smooth muscle cells^4^ (Figure 1(F)). Ca^2+^ influx into smooth muscle cells also occurs via nonselective cation channels, such as transient receptor potential channels activated by muscarinic stimulation (as shown by gene deactivation in mice).^19^ Transient receptor potential channels are voltage-independent and are activated by either intracellular Ca^2+^ or by G-protein coupled receptors.^57^ Following an increase in intracellular Ca^2+^, Ca^2+^ binds to calmodulin (ubiquitous calcium-binding protein), which activates myosin light chain kinase and subsequent phosphorylation of myosin light chain kinase, which causes smooth muscle contraction.^51^ In contrast, dephosphorylation of myosin light chain kinase results in muscle relaxation.^51^ Muscle relaxation is also controlled by cAMP/PKA signaling pathway (reviewed in details in^2^).

Blakeney et al. demonstrated that the gut microbiota-produced branched-chain fatty acid, isovaleric acid, caused relaxation of colonic smooth muscles in mice via cAMP/PKA pathway using an *ex vivo* organ bath set-up and dispersed colonic muscle cells^8^ (Figure 1(G);discussed also in section *Short-chain fatty acids*). This microbiota-dependent relaxation was found to be concentration-dependent and to occur in whole colonic segments or in isolated colonic muscle cells.^8^ Other studies focusing on the effects of gut bacteria on the intestinal smooth muscle contractility either in human or rat colonic tissue observed inhibitory effects on the gut contractility.^58,59^ Overall, though the key role of the gut microbiota and its metabolites in regulating gastrointestinal smooth muscle cells starts to be unraveled, the molecular mechanisms by which the gut bacteria act remain largely unknown.

### Gut bacterial signaling to interstitial cells

Interstitial cells of Cajal (ICCs) are the intestinal pacemaker cells responsible for the generation of electrical oscillatory activity (also known as the “slow waves”). Electrical oscillatory activity is the dominant omnipresent pacemaker activity of the intestine, that is electrically coupled to smooth muscle cells, providing it with cyclic changes in excitability.^60^ ICCs constitute the basis for peristalsis, which is then stimulated by migrating motor complexes (MMCs; intestinal motility pattern of the interdigestive state) or segmentation motility patterns (see section *Contractility and flows* below),^2,60–62^ and were first characterized by Cajal in 1893.^63^ The functional development of ICC networks depends on signaling via the Kit receptor pathway.^64^ In fact, mice with loss-of-function in c-Kit signaling failed to generate pacemaker activity^64^ indicating that ICCs serve as pacemakers.

Remarkably, a cocktail of four different lactic acid bacteria (*Lactobacillus plantarum* 2362, *Lactobacillus casei* ssp. *paracasei* 19, *Leuconostoc raffinolactis* 23 ~ 77:1, and *Pediococcus pentosaceus* 16:1), Synbiotic2000^TM^, fed to a traumatic brain injury (TBI) mouse model, which is prone to suffer from gastrointestinal motility abnormalities, was reported to improve the reduced contractile amplitudes, frequencies and tension forces in the small intestine of these mice compared to the control group.^65^ The TBI model also showed severe impairments in the number of ICCs and c-Kit protein concentrations. However, after administration of Synbiotic2000^TM,^ the number of ICCs and c-Kit protein concentrations were significantly improved, though the concentrations did not reach the levels detected in the control group.^65^ Analogously, Sui et al. showed that the probiotic strain, *Clostridium butyricum*, promoted ICCs (isolated from murine small intestine) proliferation by their regulation of the TLR2 expressed on ICCs^66^ (Figure 1(H)). Another study suggested that signaling through TLR4 by LPS, activated murine ICCs to produce nitric oxide, and thus inhibited the pacemaker currents^67^ (Figure 1(I)). ICCs represent a potentially valuable therapeutic target. Further characterization of the ICCs cells and their interactions with the gut microbes and their metabolites may provide new insights into development of novel therapies for functional gastrointestinal disorders.

### Modulation of bowel motility by bacterial-dependent production of gut hormones

Gut hormones are released from specialized intestinal epithelial cells, enteroendocrine cells, in response to meal-related stimuli. Subsequently, these gut hormones exert actions ranging from the local control of gut motility, to the regulation of glucose homeostasis, and food intake.^68^ Gastrointestinal motility is modulated by gut hormones during both the interdigestive (i.e. between meals; motilin and ghrelin), and postprandial (i.e. after meals; cholecystokinin (CCK), glucose-dependent insulinotropic polypeptide (GIP), glucagon-like peptide-1 (GLP-1), and peptide YY (PYY)) periods.^69^ GLP-1, and PYY are key mediators of the shift from an interdigestive to a postprandial gastrointestinal motor pattern.^69^ Moreover, the gut hormones CCK, GLP-1, and PYY control blood nutrient levels by modulating digestion and absorption, as slowing either of these steps reduces the rate at which ingested nutrients enter the circulation.^68^

Gut microbiota has been shown to influence the levels of gut hormones secreted into the circulatory system.^68,70,71^ For example, using fast protein liquid chromatography and liquid chromatography coupled to mass spectrophotometry, it was shown that *Akkermansia muciniphila* secretes the protein P9, which signals to a subtype of the enteroendocrine cells, L cells, to produce GLP-1^72^ (Figure 1(J)). P9-dependent GLP-1 production was associated with ameliorating thermogenesis, body weight, and glucose homeostasis in obese mice.^72^ In healthy individuals, GLP-1 induces insulin release, delays gastric emptying and increases satiety.^73^ Obese subjects had more rapid gastric emptying than their non-obese counterparts,^74^ thus GLP-1 agonists have become well-established therapies in obesity.^73,75^ Gut bacteria promoting GLP-1 production may help ameliorate obesity through reduction of gastric motility. Similarly, the production of other gut hormones was reported to be altered by the gut microbiota or their metabolites.^71,76,77^ For example, short-chain fatty acids (SCFAs, discussed in details in section *Short-chain fatty acids*), acetate, propionate, butyrate, and lactate, as well as bacterial supernatants of the *Bifidobacterium* and *Lactobacillus* genera, were shown to affect ghrelin signaling via internalization of ghrelin receptor stably expressed in human embryonic kidney cell line^71^(Figure 1(K)). In addition, physiological concentrations of propionate and butyrate were shown to induce PYY gene expression and production in human enteroendocrine cells^76^ (Figure 1(L)).

### Gut bacteria, serotonin, and control of the gut motility

It is well accepted that the greatest quantity of serotonin (5-HT) in the human body is synthesized within a subtype of the enteroendocrine cells, enterochromaffin cells, in the intestinal mucosa, via the enzyme tryptophan hydroxylase 1 (TPH1).^78^ Enterochromaffin cells act as sensory transducers to release 5-HT in response to various mechanical and chemical stimuli.^79^ In 2017, enterochromaffin cells were recognized as specialized stimulus detectors that constitute a direct line of communication between the mouse gut epithelium and enteric nervous system.^80^ A year later, it was shown that, throughout the mouse gut, enterochromaffin cells release 5-HT in response to their sensing of nutrients and microbial metabolites, indirectly through the release of GLP-1 by neighboring cells.^81^

On a much smaller scale, 5-HT is also synthesized in approximately 1% of nerve cell bodies in the enteric nervous system, via a different enzyme called tryptophan hydroxylase 2 (TPH2).^82^ Despite the low levels of exogenous 5-HT (5-HT produced in the enteric nervous system), its role in stimulating gastrointestinal motility is well established.^83^ In contrast, the role of endogenous 5-HT (5-HT produced from enterochromaffin cells) has been very challenging to resolve with many contradicting reports.^84–86^ Several studies have shown that when TPH1 is either deleted in mice or pharmacologically inhibited, it alters the murine total gut transit time, gastric emptying, small bowel propulsion, or rate of expulsion of beads inserted into the rectum.^84,85^ Analogously, *in vitro*, the colonic emptying time, propulsion of fecal pellets and migrating motor complexes were compromised in mice lacking mucosal 5-HT (Tph1^−/−^ mice).^86^ In contrast, *in vivo* studies on gut motility in the absence of the TPH1 did not lead to any inhibitory effects on the gastrointestinal transit in conscious mice.^84,85^ Therefore, it was suggested that while endogenous 5-HT is released in response to a number of stimuli and plays an important role in paracrine and endocrine systems,^87,88^ it acts only as a modulator, not as an initiator, of neurogenic motor patterns and gut transit.^89^

Numerous studies have reported that gut microbiota or their metabolites can regulate host serotonin biosynthesis from the enterochromaffin cells and thus may be involved in modulation, but not initiation of, gut motility. For example, Yano et al. showed that TPH1 inhibition in mice blocks the ability of the microbiota to promote colonic 5-HT production from enterochromaffin cells suggesting that the gut microbiota, specifically spore-forming microbes dominated by Clostridial species, requires host TPH1 activity to induce 5-HT synthesis^90^ (Figure 1(M)). Another study showed that heat-killed *Lactobacillus casei* subsp. *casei* has a potential to promote serotonin biosynthesis in the murine colonic mucosa.^91^ Employing 5-HT quantification in the colonic tissues and bead expulsion measurements after administration of heat-killed *Lactobacillus casei* subsp. *casei* showed significantly higher levels of 5-HT in the colonic tissue and significantly lower bead expulsion time. These data suggestthat promoting 5-HT synthesis could contribute to improving the bowel motility.^91^ Besides the role of TPH1 in the induction of 5-HT synthesis, 5-HT release is highly dependent on Ca^2+^ influx into the cells and enterochromaffin cells are endowed with L-type voltage-dependent Ca^2+^ channels.^92^In fact, the microbiota-produced metabolite 5-hydroxyindole, was shown to induce the production of 5-HT from an *in vitro* cell model of enterochromaffin cells, potentially, via the induction of L-type voltage-dependent Ca^2+^ channels and Ca^2+^ influx into the cells^4^ (Figure 1(M)). Taken together, the currently available data suggest that gut microbial luminal stimuli can promote 5-HT release, however what exact role the endogenous 5-HT plays in the modulation of gut motility remains to be resolved.

## Gut bacteria regulate the bowel motility via their metabolic products

One of the different ways by which gut microbiota can influence the gut motility is via the release of metabolites or end products of bacterial fermentation (Table 1). Two main groups of bacterial products, which have been well studied in relation to altered gut motility, are SCFAs, and tryptophan metabolites. In addition, several other metabolites that belong to a wide range of chemical groups have been shown to alter gut motility. Nevertheless, we are only dealing with the tip of the iceberg as there are many more microbiota-related metabolites yet to be uncovered and investigated for their potential role in regulating the gut motility.

**Table 1.** Overview of currently known bacterial metabolites, their effect on gastrointestinal motility, and experimental models used for the assessment of gut motility

Bacterial metabolite	Effect on gut motility	Methods used	Model organism and effect size (N)	Reference
Lipopolysaccharides	-Regulation of BMP2 (growth factor produced by macrophages, which regulate peristaltic activity of the colon) and CSF-1 expression (a growth factor secreted by enteric nervous system)	*-Ex vivo* organ bath model (colonic contractility measurements) Bead expulsion test (colonic transit analysis) -GI transit assay using carmine red *in vivo* (gut motility measurements) -Rhodamine B dextran fluorescence detection (gastric emptying and small intestinal transit)	Mice (N = NS*)	^40^
	-Signaling via TLR4 receptor leads to delayed gastrointestinal motility	-Fecal pellets collection (stool frequency) Bead expulsion test (colonic transit analysis) -Isometric muscle recordings of colonic longitudinal muscle strips	Mice (N = 5-10)	^36^
	-Signaling through TLR4 activates ICCs to produce nitric oxide and inhibits the pacemaker currents of the gut contractility	-Whole-cell patch clamp (cultured ICCs measurements of membrane currents and potentials) -RT-PCR in cultured ICCs from small intestine	Mice (N = 6-11)	^67^
Lipopeptides and peptidoglycan	-Signaling via TLR2 receptor resulted in inhibition of neurogenesis leading to significant dysmotility and loss of colonic myenteric neurons	-GI transit assay using carmine red *in vivo* (gut motility measurements) -Bead latency test (distal colonic transit time) -Detection of fluorescein isothiocyanate-dextran (determination of colonic geometric center)	Mice (N = NS)	^44^
Salmonella typhimurium-derived enterotoxin	-Causing dramatic changes in intestinal myoelectric activity and substantial fluid production	*-Ex vivo* organ bath (measurement of myoelectric activities in ileal loops)	Rabbits (N = 8)	^49^
Short-chain fatty acids	-Stimulation of PYY production in human enteroendocrine cells	-ELISA (PYY quantification)	NA	^76^
	-Modulation of 5-HT release from model of enterochromaffin cells (butyrate, propionate)	-RIN14B cell line *in vitro* (5-HT release measurements)	NA	^90^
	-Increase of the proportion of cholinergic neurons translating to increased gut motility	*-Ex vivo* organ bath model (colonic contractility measurements) -Bead expulsion test (colonic transit analysis)	Rats (N = 5-6)	^41^
	-Stimulation of increase/decrease in colonic motility (butyrate, propionate, acetate)	*-Ex vivo* organ bath model (colonic contractility measurements)	Guinea pigs and rats (N = 4-9 and N = 4-6)	^93,94^
	-Stimulation of GLP-1 production (butyrate, propionate, acetate)	*-Ex vivo* organ bath model (colonic contractility measurements) -Evans Blue dye detection (small intestinal transit)	Mice (N = 4-7)	^95^
	-Modulation of ghrelin signaling (acetate, propionate and butyrate)	-Cell culture (activation of G protein coupled receptors using β-arrestin assay)	NA	^71^
Tryptophan metabolites	-Acceleration of gastrointestinal transit by activation of epithelial 5-HT_4_ receptor in the proximal colon (tryptamine)	-Ussing Chamber (assessment of epithelial ionic flux) -GI transit assay using carmine red *in vivo* (gut motility measurements)	Mice (N = 4-6)	^5^
	-Modulation of secretion of GLP-1 (indole)	-Total GLP-1 assay (GLP-1 quantification)	NA	^96^
	-AhR signaling in the colonic neurons alters gut motility (indole-3-carbinol)	-GI transit assay using carmine red *in vivo* (gut motility measurements) -Live video imaging and spatiotemporal mapping of colonic motility	Mice (N = 8-18)	^39^
	-Enhancement of the epithelial barrier functions by increasing of expression of genes involved in maintenance of epithelial cell structure and function (indole)	-Microarrays	NA	^97^
	-Regulation of intestinal barrier function *in* *vivo* by acting as a ligand for xenobiotic sensor, pregnane X receptor (IPA)	-Fluorescein isothiocyanate-dextran detection in serum (intestinal permeability assay *in vivo*)	Mice (N = 3-6)	^98^
	-Reduction of intestinal permeability in mice fed a high fat diet	-Fluorescein isothiocyanate-dextran detection in plasma (intestinal permeability assay *in vivo*) -TEER (colonic intestinal permeability assay *in vitro*)	Mice (N = 7-9)	^99^
Bile acids	-Modulation of 5-HT release from model of enterochromaffin cells (cholate, deoxycholate)	-RIN14B cell line *in vitro* (5-HT release measurements)	NA	^90^
	-Bacterial bile salt hydrolase activity is associated with faster gastrointestinal transit in gnotobiotic mouse model	-GI transit assay using carmine red *in vivo* (gut motility measurements)	Mice (N = 5-6)	^100^
	-Promote gastrointestinal motility by activation TGR5 receptors located on enterochromaffin cells	*-Ex vivo* organ bath model (colonic contractility measurements) -Evans Blue dye detection (gastrointestinal transit) -Bead expulsion test (colonic transit analysis) -Fecal pellets collection (stool frequency)	Mice (N = NS)	^101^
5-hydroxyindole	-Modulation of gut motility via L-type voltage-dependent Ca^2+^ channels located on the colonic smooth muscle cells -Control of serotonin release from model of enterochromaffin cells	-RIN14B cell line *in vitro* (5-HT release measurements) -GI transit assay using carmine red *in vivo* (gut motility measurements) *-Ex vivo* organ bath model (colonic contractility measurements)	Rats (N = 6-10)	^4^
Protein P9	-Production of protein P9 signals to L cells to produce GLP-1	-ELISA (GLP-1 quantification)	NA	^72^
Indole and indole-3-carboxaldehyde	-Activation of TRPA1 in EECs, leads to production of 5-HT from enterochromaffin cells and thus modulate gut motility	-Real-time measurements of EECs *in vivo* in zebrafish (activation of TRPA1 and gut motility) -Amperometry (5-HT release)	Zebrafish (N = 117-213)	^102^
Quercetin	-Improvement of the symptoms of constipation in rat loperamide-induced constipation model	-Charcoal propulsion test (gut motility)	Rats (N = 3)	^103^
Heptadecanoic and stearic acid (saturated long-chain fatty acids)	-Enhancement of colonic contractility *ex vivo* and stool frequency *in vivo*	*-Ex vivo* organ bath model (colonic contractility measurements) -Fecal pellets collection (stool frequency)	Rats (N = 6-8)	^104^
Isovaleric acid (branched-chain fatty acids)	-Causes contractile relaxation of colonic smooth muscles via cAMP/PKA pathway	*-Ex vivo* organ bath model (colonic contractility measurements) -Isolated muscle cells culture (direct activation of PKA activity)	Mice (N = 4-7)	^8^
Polyamines (spermidine, putrescine, spermine) and trace amines (isoamylamine, cadaverine)	-Modulation of intestinal peristalsis	*-Ex vivo* organ bath model (ileal and colonic contractility measurements)	Mice (N = 7)	^105^
Ferulic acid	-Acceleration of gastrointestinal transit and gastric emptying	-Charcoal propulsion test (gut transit) -Phenol red detection (gastric emptying)	Rats (N = 8)	^106^
Histamine	-Promotion of colonic motility via activation of histamine receptors in the gut	-Fecal output assay	Mice (N = 3-5)	^107^
3-(3,4-dihydroxyphenyl)propionic acid (DHPPA)	-Potent stimulation of ileal motility *ex vivo*	*-Ex vivo* organ bath model (ileal contractility measurements)	Mice (N = 4-6)	^108^
Dopamine	-Inhibition of longitudinal muscle contractility	*-Ex vivo* organ bath model (longitudinal ileal muscle contractility measurements)	Guinea pigs (N = 10)	^109^
	-Decreased the duration of the MMCs in the small intestine (duodenum and jejunum)	-Implanted Ni/Cr electrodes	Dogs (N = 4)	^110^
	-Induced phase-III like MMCs in the duodenum	-Intestinal radiopaque tube	Humans, healthy (N = 14)	^111^

*NS = not specified **NA = not applicable.

### Short-chain fatty acids

SCFAs, including, acetate, butyrate, and propionate^112^ originate from bacterial degradation of dietary fibers and are key energy source and signaling molecules in the mammalian colonocytes.^113,114^ SCFAs, in particular, butyrate, have been shown to modulate the activity of the enteric nervous system in rats.^41^ A resistant starch diet (in which starch reaches the colon and can be considered as a dietary fiber), intracecal butyrate infusion, and butyrate application to cultured myenteric ganglia were all shown to affect the rat enteric nervous system by increasing the proportion of cholinergic neurons translating to shorter colonic transit time and increased cholinergic-mediated colonic circular muscle contractile response.^41^ Hurst et al. showed that different SCFAs have different effects on the colonic contractility in guinea pigs.^93^ Butyrate caused an increase in colonic contractility, while propionate, and, to a lesser extent, acetate resulted in a decrease in contractility.^93^ Such a difference in the effect of the different types of SCFAs studied may lie in the different animal models used in the study since previous findings reported that, in the rat colonic tissue, all three SCFAs had stimulatory effects on the colonic peristalsis.^93,94^ Furthermore, butyrate, propionate and acetate, can indirectly affect the murine gut motility through their induction of colonic GLP-1 secretion (Figure 1(J)), which was reported to significantly prolong intestinal transit.^95^

Besides the main SCFAs, branched SCFA, isovaleric acid, has been shown to have an inhibitory effect on the murine colonic contractility.^8^ Isovaleric acid was shown to act directly on the isolated colonic smooth muscles *in vitro* and to cause muscle relaxation via the PKA pathway (Figure 1(G)).^8^ Overall, bacterial-produced SCFAs are key players in the regulation of gut motility.^112,113^ However, when the levels of SCFAs are chronically elevated, they may result in a detrimental increase in colonic transit rate, which could play a role in pathogenesis, where gut dysfunction is a comorbidity, as in irritable bowel syndrome.^115^

### Tryptophan metabolites

Tryptophan metabolites generated exclusively by the gut microbiota are key contributors to the intestinal homeostasis.^46^ A growing number of evidence has associated various tryptophan metabolites with gut motility.^116^ Gut microbes can break down tryptophan to produce a variety of metabolites. For example, *Clostridium sporogenes* and other gut bacteria, such as *Ruminococcus gnavus*, harboring the tryptophan decarboxylase enzyme can produces tryptamine.^117^ Tryptamine was recently shown to accelerate gastrointestinal transit by activating epithelial serotonin receptor 4 (Figure 1(N)), and by increasing the anion-dependent fluid secretion in the proximal colon of mice.^5^ Moreover, both tryptophan and tryptamine were significantly increased in fecal samples from diarrhea-predominant irritable bowel syndrome (IBS-D) patients, suggesting that these metabolites might be responsible for the higher water content of feces in IBS-D.^118^ Another gut bacterial metabolite of tryptophan is indole, which is produced by gut bacteria harboring the tryptophanase enzyme.^119^ Indole has been observed to modulate the secretion of GLP-1 from immortalized and primary mouse colonic enteroendocrine L cells^96^ (Figure 1(J)). Indole, as well as another bacteria-derived tryptophan metabolite, indole-3-carboxaldehyde, produced by *Edwardsiella tarda*, were recently shown to activate the transient receptor potential ankyrin A1 (Trpa1) in enteroendocrine cells of zebrafish, mouse and human, and to induce the production of 5-HT (Figure 1(M)), thus accelerate intestinal motility.^102^ As mentioned above, AhR signaling has also been recently associated with the regulation of intestinal transit time in mice^39^ (Figure 1(E)). Bacterial-produced tryptophan metabolites, such as indole, tryptamine, skatole, indoleacetic acid, indoleacrylic acid, indole-3-carboxaldehyde and indolelactic acid are widely described as ligands of AhR,^46^ thus may be involved in the control of bowel motility. Moreover, indole was shown to enhance the intestinal epithelial barrier function in human colon-cancer cell line and *in vivo* in mice studies by increasing the expression of genes involved in maintenance of epithelial cell structure and function.^97,120^ Impaired intestinal permeability, which is a significant factor in several (gastrointestinal) diseases, such as inflammatory bowel disease, irritable bowel syndrome, celiac disease or colon carcinoma, has been linked to dysmotility.^121^ The intestinal barrier prevents loss of water and electrolytes and entry of antigens and microorganisms into the body, while allowing exchange of molecules between host and environment and absorption of nutrients in the diet.^121^ Therefore, normal functioning of gut barrier is necessary for healthy gastrointestinal transit. Similarly, indolepropionic acid (IPA) another bacterial metabolite of tryptophan produced via the phenyllactate dehydratase gene cluster^122^ was found to regulate the intestinal barrier function *in vivo* in mice by acting as a ligand for the xenobiotic sensor, pregnane X receptor, particularly in the presence of indole.^98^ In addition, IPA reduced intestinal permeability in mice fed a high fat diet.^99^ Lastly, another bacterial-produced indole derivative, 5-hydroxyindole, was shown to be a potent stimulant of rat intestinal contractility via its action on L-type Ca^2+^ channels (see also section *Bacterial interaction with the gastrointestinal smooth muscles*) (Figure 1(F)).^4^ 5-hydroxyindole is a structural analogue of indole and is produced by bacterial degradation of 5-hydroxytryptophan, which is a chemical precursor and intermediate metabolite in the biosynthesis of 5-HT.^4^

### Other bacterial metabolites

**Figure 2.**
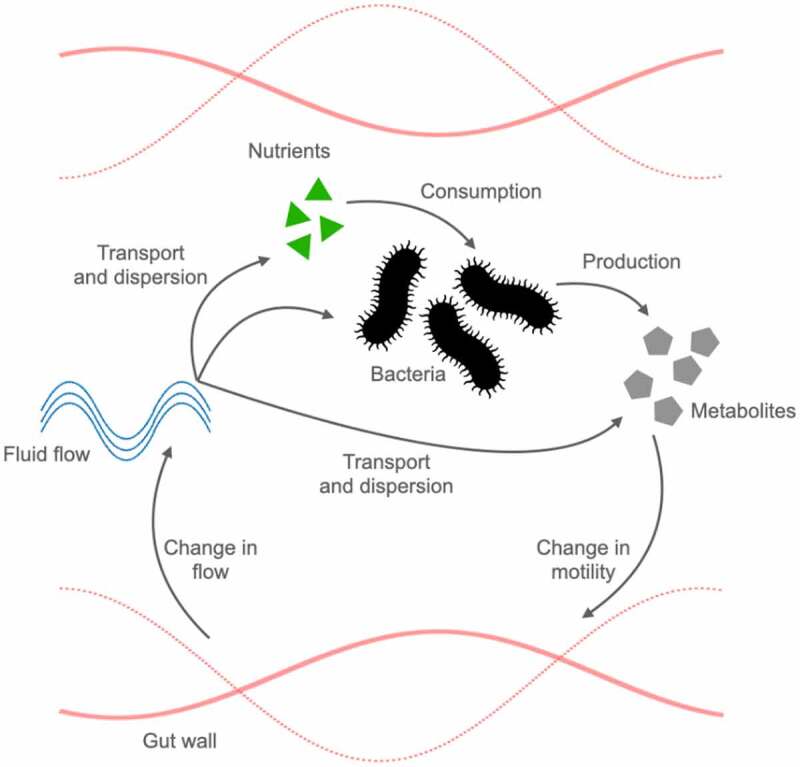
A comprehensive *in silico* model of bacterial-produced metabolites and motility interactions. Bacterial-produced metabolites can directly influence gut motility, leading to a cascade of effects and feedback mechanisms impacting the production of the metabolite itself. Gut bacteria replicate and produce their metabolites based on the availability of nutrients. Metabolites impact gut motility, which, in turn, modifies the fluid flow. The flow is directly responsible for the transport and dispersion of nutrients, bacteria, and their metabolites, closing the feedback loop.

Studies on the effect of nutrients on regulating the gut motility have unraveled numerous compounds to affect the gut motility, several of which are the products of gut bacterial metabolization of these nutrients. For example, quercetin, an abundant flavonoid found in many fruits, vegetables and grains,^123^ but also produced by gut bacteria, specifically Fusobacteria species^124,125^ can improve the symptoms of constipation in rat loperamide-induced constipation model. The laxative effects of quercetin have been attributed to the interaction between quercetin and muscarinic receptor signaling pathway.^103^ Saturated long-chain fatty acids, heptadecanoic acid and stearic acid, produced by gut bacteria, specifically *Prevotella, Lactobacillus* and *Alistipes* genera, were observed to enhance the rat colonic contractility *ex vivo* and defecation frequency *in vivo*.^104^ Another study showed that polyamines (spermidine, putrescine, spermine) and trace amines (isoamylamine, cadaverine) derived from intestinal bacteria may act as chemosensors and thus modulate the rat intestinal peristalsis.^105^ Bile acids affect several gastrointestinal functions including absorption, gastric emptying, and small intestinal and colonic motility in mice.^100^ Bile acids are modified by gut microbiota and the bacterial bile salt hydrolases activity was correlated with faster gastrointestinal transit in gnotobiotic mouse model.^100^ Moreover, that study showed that commonly used spice, turmeric, affects gut motility through bacterial bile salt hydrolase activity and Ret signaling (*Ret* is a gene implicated in Hirschsprung’s disease, a development disorder associated with absent peristalsis in the distal colon) in the enteric nervous system.^100^ Deoxycholate is a secondary bile acid, produced by microbial biotransformation of cholate and was reported to inhibit the murine spontaneous contractility of colonic longitudinal muscle by mechanism that requires expression of TGR5 G protein-coupled receptors on enterochromaffin cells^101^ (Figure 1(M)). Furthermore, ferulic acid, a potent anti-inflammatory and antioxidant compound was shown to be produced by *Lactobacillus fermentum*^126^ and to accelerate the gastrointestinal transit and gastric emptying in a dose-dependent manner in rats.^106^ Another study showed that a shift in colonic metabolism from carbohydrate fermentation to protein catabolism, as reflected by higher urinary levels of potentially deleterious protein-derived metabolites, is associated with longer colonic transit time in humans.^127^ Additionally, gut microbiota is able to produce neurotransmitters involved in gut motility, such as γ-aminobutyric acid (GABA).^128,129^ GABA receptors are expressed in the enteric nervous system, where GABA may modulate intestinal motility.^130^ Lastly, the dietary histidine has been observed to be metabolized by *Morganella morganii* and *Lactobacillus reuteri* strains into histamine, which significantly increases fecal output (number of fecal pellets per hour) via activation of histamine receptors along the murine gut.^107^ Collectively, the interaction between dietary components and gut microbiota is a key element in the regulation of gut motility, which in turn, determines which microbes colonize the gut,^131^(see section *Design of in silico studies to unravel the microbiota-gut motility interplay* and Figure 2 below), entering a vicious circle.

Besides the ability of gut microbiota to take up dietary small molecules, they can also sequester medical drugs with consequences on interpersonal variation in drug efficacy and toxicity.^132^ For example, we showed that levodopa, the primary treatment of Parkinson’s disease, in particular, its unabsorbed residues, is deaminated by *C. sporogenes* to 3-(3,4-dihydroxyphenyl)propionic acid (DHPPA), a potent inhibitor of mouse ileal contractility *ex vivo*.^108^ Moreover, bacterial species, such as *Enterococcus faecalis, Enterococcus faecium* and *Lactobacillus brevis*, can produce luminal dopamine.^133^ Dopamine has been shown to affect gut motility in several model organisms, including rodents, dogs and humans.^109–111^ For example, in guinea pigs, it was shown that dopamine inhibits longitudinal muscle contractions.^109^ In dogs, dopamine decreased the duration of the migrating motor complexes in the small intestine 1 h prior a meal compared to controls^110^ and in fasted human subjects, intravenous administration of dopamine induced phase-III like migrating motor complexes (last phase in the MMC cycle, which consists of strong contractions to completely occlude the lumen) in the duodenum.^111^ This, and similar studies, underpin the importance of the metabolic pathways of the gut microbiome involved in drug metabolism not only to preserve drug effectiveness, but also to avoid potential side effectsof bacterial breakdown products of the unabsorbed residue of medication, such as inhibition of gut motility.

## Models used to decipher the mechanisms underlying bacterial regulation of the gut motility

Deciphering the mechanisms by which gut bacteria and their metabolites influence gut motility is crucial to understand the role of these microbes in bowel dysfunction and its targeting by the design of microbiota-based interventions. To date, experimental research investigating the effect of microbiota on gut motility is directed along three main types of methodologies; *in vivo* human or animal studies,^4–7^ *in vitro* and *ex vivo* experiments (Tables 1 and 2), the latter two focus on unraveling the mechanisms underlying the observations made *in vivo*.^4,5,8^ *In vivo* studies in humans and animals include magnetic resonance measurements, manometry measurements, mini-capsules or ingestion of surrogate markers, such as carmine red dye or fluorescein isothiocyanate.^4,99,136–139^ These measurements allow the functional scanning of gut motility (e.g., water flux and water content),^136^ the acquisition of intraluminal pressure patterns,^137,139^ the determination of gastrointestinal transit time,^4,140^ as well as intestinal permeability.^99^ Transit time can also be measured with other techniques, such as evaluation of the Bristol Stool-Scale score^135^ and recently, a new method for gut transit time measurements in humans was introduced, namely the blue dye method.^134^ The blue dye method provides a novel, inexpensive and scalable assessment of gut transit time compared to traditional measures of stool frequency.^134^

**Table 2.** Overview of gut bacterial taxa currently described to impact gastrointestinal motility, and experimental models used for motility assessment

Gut bacterial species	Effect on gut motility	Methods used	Model organism and effect size (N)	Reference
*-Lactobacillus acidophilus* *-Bifidobacterium bifidum*	-Administration of *L. acidophilus* and *B. bifidum* to germ-free rats displayed improved intestinal transit and contractility of the small intestine	-Myoelectric recording *in vivo* -Measurements of a radioactive marker (Na_2_^51^CrO_4_) distribution along the small intestine	Rats (N = 5-18)	^35^
*-Bacteroides thetaiotamicron*	-Critical for enteric nervous system innervation -Administration of *B. thetaiotamicron* (*Bt*) restored the expression of excitatory and inhibitory motor neurons signaling enzymes	-Colonic manometry *in vitro* (colonic motility measurements in the colonic tissue of SPF, GF and *Bt*-conventionalized mice) -Immunohistochemistry (neuronal cell populations determination) -qPCR (expression of ENS signaling enzymes)	Mice (N = 3-5)	^42^
*-Vibrio cholerae*	-Use of the syringe-like type VI secretion system for induction of intestinal movements, which leads to expulsion of the resident microbiota by the host	*-In vivo* imaging of fluorescently-labeled bacterial populations and dynamics of unlabeled intestinal tissue (motility)	Zebrafish (N = 5-6)	^48^
*-Lactobacillus rhamnosus GG*	-Increase of choline acetyltransferase expression (responsible for synthesis of main metabolite involved in gut motility, acetylcholine) in the ENS after administration of *L. rhamnosus* GG	-RT-PCR -Immunoblotting -Immunostaining	Mice (N = 3-5)	^43^
*-Escherichia coli* Nissle 1917	-Inhibitory effect on the smooth muscle contractility	*-Ex vivo* organ bath model (contractility measurements after application of *E. coli* Nissle 1917 bacterial supernatant)	Rats (N = 5)	^59^
*-Bifidobacterium longum* *-Lactobacillus acidophilus* *-Streptococcus thermophilus* *-Enterococcus faecalis*	Inhibitory effect on human colonic smooth muscle *in vitro*	*-Ex vivo* organ bath model (contractility measurements after application of sonicated cell fractions and bacterial supernatants)	Humans (N = 25)	^58^
*Akkermansia muciniphila Bacteroides spp* *-Alistipes*	Modulation of longer gut transit time in humans	-Blue dye method (measurements of gut transit time in humans)	Humans (N = 863)	^134^
*-Ruminococcus -Bacteroides* *-Prevotella*	-Abundance of *Ruminococcus* and Bacteroides is linked to shorter transit times -Abundance of Prevotella is linked to longer transit times	-Bristol Stool Scale score (measurements of colonic transit times in humans)	Humans (N = 53)	^135^
-Lactic acid bacteria (*Lactobacillus plantarum 2362, Lactobacillus casei ssp. paracasei 19, Leuconostoc raffinolactis 23~77:1, and Pediococcus pentosaceus 16:1*)	-Amelioration of the small intestinal contractile impairment in traumatic brain injury mouse model fed with probiotic mixture	*-Ex vivo* organ bath model (contractility measurements)	Mice (N = 6)	^65^
*-Clostridium butyricum*	-Promotion of ICCs proliferation and intestinal motility by the regulation of TLR2 expression on ICCs after stimulation with *C. butyricum* suspensions	-Cell culture (culture of ICCs) RT-PCR (expression of TLR2) -Western Blot (protein levels of TLR2)	NA	^66^
*-Bifidobacterium -Lactobacillus*	-Modulation of ghrelin signaling (acetate, propionate and butyrate)	-Cell culture (activation of G protein coupled receptors using β-arrestin assay stimulated with bacterial supernatants)	NA	^71^
-Spore-forming bacteria	-Modulation of metabolites that promote transit time	-GI transit assay using carmine red *in vivo* (gut motility measurements after colonization of germ-free mice with fecal samples from spore-forming conventionalized mice)	Mice (N = 4-8)	^90^
*-Lactobacillus casei* subsp. *casei*	-Administration of heat killed *L. casei* subsp. *casei* increases levels of 5-HT in the colonic tissue and lowers bead expulsion time	-HPLC (5-HT levels) Bead expulsion test (colonic transit analysis)	Mice (N = 6)	^91^
*-Escherichia coli -Fusobacterium nucleatum*	-Modulation of gut motility via L-type voltage-dependent Ca^2+^ channels located on the colonic smooth muscle cells -Control of serotonin release from model of enterochromaffin cells	-RIN14B cell line *in vitro* (5-HT release measurements after application of 5-hydroxyindole produced from *E. coli* and *F. nucleatum* derived)) -GI transit assay using carmine red *in vivo* (gut motility measurements after application of 5-hydroxyindole produced from *E. coli* and *F. nucleatum* derived) *-Ex vivo* organ bath model (colonic contractility measurements after application of 5-hydroxyindole produced from *E. coli* and *F. nucleatum* derived-)	Rats (N = 6-10)	^4^
*-Akkermansia muciniphila*	-Production of protein P9 signals to L cells to produce GLP-1	-ELISA (GLP-1 quantification after stimulation with bacterial pellets or supernatants)	NA	^72^
*-Edwardsiella tarda*	-Activation of TRPA1 in EECs, leads to production of 5-HT from enterochromaffin cells and thus modulate gut motility	-Real-time measurements of EECs *in vivo* in zebrafish (activation of TRPA1 and gut motility after oral gavage of indole or indole-3-acetaldehyde produced from *E. tarda* to zebrafish) -Amperometry (5-HT release after application of indole or indole-3-acetaldehyde produced from *E. tarda* to the mouse or human small intestinal tissue)	Zebrafish (N = 117-213)	^102^
*-Fusobacteria*	-Improvement of the symptoms of constipation in rat loperamide-induced constipation model	-Charcoal propulsion test (gut motility measurements after oral administration of quercetin derived from *Fusobacteria* genera to rats)	Rats (N = 3)	^103^
*-Prevotella* *-Lactobacillus -Alistipes*	-Enhancement of colonic contractility *ex vivo* and stool frequency *in vivo*	*-Ex vivo* organ bath model (colonic contractility measurements after application of saturated long-chain fatty acids derived from *Prevotella, Lactobacillus* and *Alistipes* genera to the rat colonic tissue) -Fecal pellets collection (stool frequency measurements after conventionalization of germ-free rats)	Rats (N = 6-8)	^104^
*-Lactobacillus fermentum*	-Acceleration of gastrointestinal transit and gastric emptying	-Charcoal propulsion test (gut transit measurements after oral administration of ferulic acid derived from *L. fermentum* to rats) -Phenol red detection (gastric emptying measurements after oral administration of ferulic acid derived from *L. fermentum* to rats)	Rats (N = 8)	^106^
*-Morganella morganii -Lactobacillus reuteri*	-Promotion of colonic motility via activation of histamine receptors in the gut	-Fecal output assay (after individual mice were fed with L-histamine)	Mice (N = 3-5)	^107^
*-Clostridium sporogenes*	-Potent stimulation of ileal motility *ex vivo*	*-Ex vivo* organ bath model (ileal contractility measurements after application of 3-(3,4-dihydroxyphenyl)propionic acid derived from C. sporogenes to the ileal rat tissue)	Mice (N = 4-6)	^108^

Yet, gut bacterial distribution is not accessible *in vivo. In vitro* studies involve cell culturing techniques, such as isolation of smooth muscle cells,^8^ neuronal cell populations^42^ or ICCs.^66,67^ Among others, cell line models of enterochromaffin cells are used to study 5-HT release upon stimulation of microbial stimuli.^4,90^ Electrophysiology studies, such as whole-cell patch clamp are employed to measure membrane currents and potentials.^67^ Other molecular biology techniques such as RT-PCR, qPCR and immunohistochemistry are used in gene expression studies and detection of specific proteins in the intestinal tissue, respectively.^42,43,66,67^ *Ex vivo* organ bath systems remain the most frequently used method to unravel the effects of gut bacteria and their metabolites on gut contractility.^4,8,38,39,42,58^ An organ bath set-up employs suspending a section of intestinal tissue in a bath, oxygenated with carbogen gas mixture (5% CO_2_, balanced with O_2_) in a Krebs-Henseleit solution, and the application of a pressure transducer and data-acquisition software.^4,58,108^ The organ bath systems are designed to imitate *in vivo* situation. Although an organ bath clearly misses the biological complexity of an organism, organ bath-based studies provide the possibility to elucidate the mechanisms underlying the effects of gut bacteria or their metabolites on the intestinal contractility.^4,8,40^ In recent years, mini-guts or guts on chip^141–143^ models have emerged as very promising tools to investigate the complexities of the gut motility system. Analogously, the Ussing chamber system can be utilized as a valuable tool for measuring gut integrity and intestinal permeability,^5,117^ both of which have their impact on the gut motility as discussed earlier.

However, the complexity of the different processes that occur during digestion and absorption of nutrients, the vast interindividual variability in terms of their microbiota composition and the limitations of the currently available techniques,^137^ make gaining such a mechanistic insight very challenging. The complexity further arises from the fact that multiple physical, biological and chemical aspects interact to determine the dynamics of the gut. For example, gut bacteria, their metabolites and the nutrients on which the bacteria feed upon are transported, mixed and dispersed by the fluid flow of the gut, and thus, the stability of the bacterial population is strongly influenced by the flow. At the same time, the fluid flow is determined by gut motility, which is affected by bacterial metabolites, as well as by external factors such as the feeding state, circadian rhythm, meal content and viscosity (Figure 2). Therefore, to understand the long-term consequences of bacterial metabolites-host interactions, one has to take into account the biophysics of the gut as an interconnected system.

Currently, *in silico* models are employed to address some of the above-mentioned limitations, and focus on some of the key components of the system. For example, *in silico* models allow the integration of gut motility with an exact fluid flow calculation, solute and bacterial transport, and feedback of bacteria and components on motility and flow . These systems can accommodate exact organ geometries and motility patterns, which cannot be mimicked in an *in vitro/ex vivo* system. Moreover, such systems are flexible, and can help to explore the impact of many different, experimentally unknown, parameters in a systematic way. Three main areas of research have been addressed so far *in silico* and in theory: 1) motility and its effects on fluid flows, 2) transport and absorption of solutes, as well as digestion, and 3) bacterial population. Each of these areas is essential to build a complete picture to understand the effect of the microbiota and its metabolites on motility via the ensuing fluid flow that transport them. Despite being promising, *in silico* modeling is still in its infancy and an *in silico* model including all the interactions between motility, flow, transport, bacteria and metabolites is yet lacking. To the best of our knowledge, no *in silico* study including the feedback of bacterial metabolites onto motility has been developed yet. However, combining modeling with experimental data may lead the way to address many unresolved questions with respect to the microbiota-host interplay.^144^ Herein, we review currently available information that may help researchers in the field to structure a comprehensive model accounting for motility-induced flows, flow-associated transport of microbes and metabolites, as well as feedback of metabolites on motility (see Supplementary information for more information on on flow and transport in tubes providing background information for our review on existing *in silico* studies).

## Design of *in silico* studies to unravel the microbiota-gut motility interplay

While how bacteria regulate motility via their produced metabolites has been studied extensively, unraveling the full interplay of microbiota and gut motility also requires understanding of the feedback of gut motility on the microbiota (Figure 2). Metabolites that change motility impact flows and thus nutrients absorption, indirectly affecting the host wellbeing. Moreover, changes in the flow will impact the microbiota population and growth, and thus the metabolites produced. *In silico* models are the most indicated models to take into account all of these aspects of the microbiota-gut motility interplay. So far, a comprehensive model that combines all these aspects is still lacking. To build a comprehensive description, the first challenge is to quantitatively model the metabolite-motility interaction, which is widely unexplored in *in silico* models. Although this proves to be difficult, one can still use effective descriptions of flow and transport, as outlined above, to assess how metabolite induced-changes in motility impact the long-term distribution of bacteria and nutrients. In the following section, we outline how to describe motility, fluid flows and particle dispersion to pave the way for a comprehensive model of microbiota-gut motility interplay. We further highlight challenges in describing the metabolite-motility interaction first and foremost.

### Contractility and flows

Flows are determined by the gut motility and the gut geometry (see the Supplementary Information for more theoretical details); thus, any impact of metabolites onto motility affects the flow. One strategy for *in silico* studies to account for the effect of bacterial metabolites on motility is to employ motility patterns from experimental observation, as provided by exact motility maps (*i.e*. video recordings of the gut contractions) of *ex vivo* models,^61,62,145–147^ magnetic resonance imaging (MRI) measurements *in vivo*,^136–138^ pressure measurements *in vivo* with manometers^137,139^ or mini capsules.^137,138,140^ The observed motility patterns can be also generalized into theoretical maps from *ex vivo* motility maps.^61,62^ So far, only the most basic motility patterns have been studied, but the approach can be extended to metabolite-affected patterns. Another approach to model the effect of metabolites on gut motility is by engineering the contraction patterns using *in silico* models of the gut wall. The gut wall is viewed as an elastic or viscoelastic membrane representing the intestinal muscle layer, which is activated by the ICCs network through electrical stimuli (taken by *ex vivo* recordings), and thus produces a motility pattern.^148–151^ The underlying challenge is to understand and describe the effect of bacterial metabolites onto the ICCs network and the muscular contractions. Yet the advantage is the potential for direct comparison of simulated contraction patterns with experimental recording for model verification. To date, three types of contractions have been modeled *in silico* under normal conditions or after nutrients stimulation: peristalsis, segmentation/local stationary contraction, and pendular activity/longitudinal contractions. For the gut, multiple definitions of peristalsis have been described:^152^ the peristaltic reflex describes the bolus movement, where circular and longitudinal muscles combined, produce a constriction upward and a dilation downward;^152–154^ or a sine-like wave or a train of sine waves (as for example in the coordinated contractions of the migrating motor complexes phase III, see also the Supplementary Information)).^61,151,152,155–158^ Segmentation is also subjected to multiple definitions, from stationary local contractions induced exclusively by the circular muscles^145,154,159–163^ to Canon’s segmentation,^164^ which can be described as modulation of sine waves.^61,62^ Finally, pendular activity is connected to the contraction of the longitudinal muscles.^145,159–161,165–168^ Segmentation and pendular activity are mainly postprandial,^61,62,169^ while peristalsis happens both for the bolus transport in the peristaltic reflex,^152,169^ thus postprandial, and during migrating motor complexes, thus during fasting.^152,158^ If the bacterial metabolite-induced changes in motility correspond to or are similar to one the above-mentioned well-known patterns, then existing literature outlined above would help in modeling which type of flow is produced by the motility pattern and how it affects the host and microbiota composition. In fact, once motility is known together with the inflow within the gut, both the flow in the axial (*i.e*. along the tube) and the radial direction are determined . Radial flows impact metabolite solute mixing, its availability at the wall for absorption, and its radial distribution; longitudinal flows impact the longitudinal dispersion of the solute and its transit times, with longer transit times correlated to higher absorption.

Different types of motility have different impact on the radial and longitudinal flows. For the longitudinal flow, it was shown that peristalsis is mainly propelling with a high net longitudinal flow forward associated with cleansing,^61,151,169,170^ while segmentation and pendular activity are slowing the content down to increase absorption,^61,169^ with strong longitudinal flows forward and backward (increasing dispersion) but a low net-flow movement forward. For the colon, where absorption of water is important, pumping of water decreases the longitudinal velocity along the tube.^171^ As for radial effects, the moving walls produce radial flows promoting the solute’s radial displacement.^156,160,163,171,172^ Often vortices are reported for low viscosity environments and water-like content, although increasing viscosity decreases such vortices, so in a real system where viscosity is higher due to the food content or the presence of fibers, vortices should play a minor role.^139,153,154,157,160,163,165,167^ Vortices are connected to mixing effects^156,173^ (discussed in the next section).

Viscosity has also been discussed in relation to the mucus layer of the intestine elsewhere.^153,171,174–177^ Not only macro flow (i.e., flow across the lumen and along the tube), but also microflow due to the villi movement are considered. Villi movement produces a local flow in the fluid layer close to the wall that increases absorption and mixing.^166,172,173,178–181^ Together, the gut, by changing its motility, changes the radial flows, turbulence, mixing, and net flow forward, thus transit time. The gut can also switch between fast and slow flows varying parameters like occlusion (higher occlusion means more mixing and faster flows), wavelength and frequency (higher frequencies means higher flows).^182,183^ Thus, any impact from the bacterial metabolite on motility pattern or on motility parameters will consequently change flows, which in turn will impact nutrients and bacterial-produced metabolites diffusion, but also bacterial colonization (Figure 2).

### Transport and absorption of solutes

Describing solute transport (see Supplementary Information), secretion, degradation and absorption are essential to model the bacterial metabolite effect on motility. Solutes can be gases like oxygen or nutrients (such as glucose and amino acids), drugs, bacteria, bacterial metabolites, or antibodies. In general, solutes are diffusing, and advected by the flow, thus enhancing their apparent diffusivity due to the Taylor dispersion effect (see Supplementary Information).^184^ Solutes and particles can be mixed by the flow vortices, react with other solutes (through chemical reactions, degradation, or consumption by the microbiota), and be secreted or absorbed at the wall. To investigate the effect of fluid flows on solutes, transit times, absorption, and mixing are generally addressed. Absorption by the gut walls (for example of glucose in the small intestine) is increased if the solute remains in the gut a sufficiently long time,^173,178^ which can be achieved by slow flows thus long transient times, by long tubes, and an extended absorption surface (which is increased by the villi). Mixing and enhanced diffusion at the macro and micro levels due to flow, contractions and villi movement are also improving absorption,^173^ since the solute in this way is radially transported more efficiently to the wall. Mixing also helps to make the nutrients come in contact in the bulk with the enzymes secreted by the gut.^173^ It is well established that local circular muscle contractions, pendular activity and segmentation increase mixing and that increasing parameters such as occlusion, wavelength and frequency of contraction also increase mixing.^173^ Increasing chyme viscosity (for example, by increasing dietary fiber content) decreases mixing, since vortices which could arise in water-like environment decrease in duration and intensity or disappear,^173^ and decreases particle diffusivity (which enables particles to escape the flow streamlines and move radially to the walls for absorption, even in the absence of vortices). Accordingly, a higher content in dietary fiber can impact nutrients and drugs absorption.^138,177,185^ In conclusion, motility will impact flows and solute transport; this influences the solutes dynamics and interactions. Therefore, bacterial-produced metabolites, acting on motility, have an indirect effect on the solutes, thus on their own diffusion and advection (Figure 2). This type of feedback can determine the fate of the bacterial metabolite itself, e.g., whether the metabolite-induced motility aims at increasing or decreasing the metabolite concentration, which, in turn, affects the growth and colonization of the microbiota, and eventually impacts host health. Only by considering the impact of the fluid flow can these points be addressed.

### Bacteria and their interactions with the flow

Bacteria can be considered as particles transported and mixed by the flow, similar to solutes. Moreover, their dynamics is coupled to the nutrient and oxygen availability. Bacterial metabolites are also subjected to the same flow effects, while also being connected to the bacteria dynamics. Therefore, to assess the impact of metabolites on the gut system, the relationship between bacteria and fluid flows should be addressed. Nevertheless, there are few studies that relate flow and bacterial washout. This section focuses on i*n silico* studies which can be used as future basis to explore the flow effect on bacteria, which in turn, will help simulate the impact of bacterial-produced metabolites on motility (Figure 2). For the small intestine, Ishikawa et al. focused on the washout conditions, the influence of flow, nutrients, and oxygen availability for aerobic and anaerobic bacteria, for peristalsis only.^186^ The study showed that, even with peristaltic flow, a stable bacterial population can be found in the small intestine. Specifically, the flow field enhances the radial variation of nutrients and bacterial concentration. The authors observed a longitudinal bacterial distribution coherent with experimental data, with aerobes in higher numbers on the upstream side, and anaerobes on the downstream side. For the colon, bacterial washout was also discussed by Cremer et al. for peristalsis.^141^ Motivating their *in silico* model on mini-gut data, the authors showed, *in vitro* and *in silico*, that repeated contraction is crucial to avoid washout, even though a strong longitudinal flow is present. In fact, mixing helps to overcome flow. In their following study, Cremer et al., showed how pH and water absorption are crucial in shaping the bacterial distribution in the colon.^187^

Nonetheless, most of the *in silico* studies for the colon aim to represent digestion in the most complete way. Usually, these models include a high number of equations and parameters, describing different bacterial species, their interactions, degradation of fibers, and production of metabolites.^137,171,174,176,188–191^ In these studies, the flow is discussed mainly as an effective transit time, influenced by viscosity, the mucus layer, and water absorption. Interestingly, Labarthe et al. incorporated specific radial wall motility into the model by effective radial flows, and showed that radial gradients of viscosities due to mucus layer and water absorption, together with chemotaxis and radial flows, help the presence of bacteria at the walls: bacteria find a niche where they can prosper, helping their adhesion to the mucus layer.^171^ These bacteria can then detach and seed the lumen, contributing to the colonization of bacteria. This result calls for the need of incorporating such type of radial dynamics when describing the bacterial presence and their produced metabolites in a realistic way. Still, it is not clear how to implement different types of motility in these models, although varying longitudinal and radial mean flow intensity can be used as a proxy for different motilities.

## Conclusions

Even though the knowledge gap between the microbiota and bowel function is narrowing, enormous efforts are undoubtedly needed to fully unravel the underlying mechanisms, thereby potentiating the application of microbiota-targeted therapies in clinical practice and in a personalized manner. Future *ex vivo* and *in vivo* studies should focus on unraveling the basic mechanisms required to feed the *in silico* models. The latter should focus on the representation of the different elements that regulate the interaction between gut microbes and bowel movement, as well as feedbacks, including physics elements like fluid flow. Predictions from *in silico* and *ex vivo* can be then verified in *in vivo* models, to evaluate if all gut motility-regulatory elements are taken into consideration. Altogether, combining *in silico, ex vivo, and in vivo* outcomes will provide novel mechanistic insights in the microbiota-gut motility interplay.

## Supplementary Material

Supplemental Material
