# Supplementary material for: Gut microbiota-motility interregulation: insights from in vivo, ex vivo and in silico studies
Source: Gut Microbes. 2022 Jan 3;14(1):1997296. doi: 10.1080/19490976.2021.1997296 (PMC8741295; doi:10.1080/19490976.2021.1997296)
Supplement: Supplemental Material [file KGMI_A_1997296_SM6054.docx]

**Supplementary Information**

**Gut microbiota-motility inter-regulation: insights from *in vivo*, *ex vivo* and *in silico* studies**

**Authors:**

Barbora Waclawiková^1*^, Agnese Codutti^2*^, Karen Alim^2,3^, Sahar El Aidy^1#^

**Affiliations:**

^1^ Host-Microbe Interactions, Groningen Biomolecular Sciences and Biotechnology Institute (GBB), University of Groningen, Groningen, the Netherlands

^2^ Max Planck Institute for Dynamics and Self-Organization, Göttingen, Germany

^3^ Technische Universität München, Physics Department and Center for Protein Assemblies (CPA), Garching, Germany

^*^Shared first author

^#^Correspondence: Sahar El Aidy, email: sahar.elaidy@rug.nl

**Motility-induced fluid flow and ensuing transport**

Gut motility induces fluid flows that transport bacteria and their metabolites, thus determining their density in the gut. The physics of fluids fully defines the flow field given a motility pattern by the Navier-Stokes equation in combination with the principle of conservation of fluid volume^1^. Yet, solving for the full flow field may be computationally very expensive and it desired results are obtained faster when simplifying the description of flows by the following assumption that 1) the fluid is exhibiting a linear response to an applied force and is incompressible, i.e., not undergoing any changes in density, 2) considering the spatiotemporal complexity of motility, it is desirable to check that flow is viscous and steady^1,2^. Within these approximations, flows can be calculated as a closed and therefore quick to solve numerically expression for any motility of the tube wall^3^. Of particular interest is a motility pattern consisting of a train of traveling waves of contractions known as peristalsis^4^. Peristalsis allows to generate flows autonomously within a tube, i.e., without externally applied pressure gradient or inflow. Peristaltic flow speed depends strongly on the magnitude of the tube occlusion and wave velocity^4^ but also on the exact superposition of individual waves^5^. Due to the interplay of the flow along a peristaltic tube and the flow perpendicular to the tube axis, fluid volumes can circle within the tube, and can also travel retrograde, i.e., in the opposite direction of the peristaltic wave motion^4^. The intricate circling of flows also suggests that peristalsis can be used to mix fluids^6,7^.

Motility-induced flows transport minute particles of any substances dissolved within the fluid. The challenge here is that the transport of such minute particles, which could be bacteria, metabolites, nutrients, signaling molecules or reactants of any kind is governed not only by the streamlines of the flow but also by the erratic diffusion of the particles. Albeit being a very slow process, diffusion is very impactful as it drives particles to hop in between different streamlines, thereby accelerating their journey when arriving on a streamline of larger velocity or slowing down if the particle diffused onto a slower streamline. This effect grows with the spatial gradient in flow velocities. Direct solutions for the trajectory of a particle is again computationally very expensive, as is solving for the full dynamics of a concentration of particles^8^. Here, computational time can be saved if the concentration of particles quickly averages out across the cross-section of a tube. Then, spreading dynamics can be described for the cross-sectional averaged concentration by an effective velocity and an effective diffusivity (the so called Taylor dispersion)^9,10^.

**References**

1. Batchelor, G. K. *An Introduction to Fluid Dynamics*. *An Introduction to Fluid Dynamics* (Cambridge University Press, 2000).

2. Womersley, J. R. Oscillatory flow in arteries: The constrained elastic tube as a model of arterial flow and pulse transmission. *Phys. Med. Biol.* **2**, 178–187 (1957).

3. Li, M. & Brasseur, J. G. Non-Steady Peristaltic Transport in Finite-Length Tubes. *J. Fluid Mech.* **248**, 129–151 (1993).

4. Shapiro, A. H., Jaffrin, M. Y. & Weinberg, S. L. Peristaltic pumping with long wavelengths at low Reynolds number. *J. Fluid Mech.* **37**, 799–825 (1969).

5. Baüerle, F. K., Karpitschka, S. & Alim, K. Living System Adapts Harmonics of Peristaltic Wave for Cost-Efficient Optimization of Pumping Performance. *Phys. Rev. Lett.* **124**, 098102 (2020).

6. Selverov, K. P. & Stone, H. A. Peristaltically driven channel flows with applications toward micromixing. *Phys. Fluids* **13**, 1837–1859 (2001).

7. Arrieta, J. *et al.* Geometric mixing, peristalsis, and the geometric phase of the stomach. *PLoS One* **10**, e0130735 (2015).

8. Risken, H. Fokker-Planck Equation. in *The Fokker-Planck Equation Methods of Solution and Applications* 63–95 (Springer, Berlin, Heidelberg, 1996).

9. Taylor, G. Dispersion of soluble matter in solvent flowing slowly through a tube. *Proc. R. Soc. London. Ser. A. Math. Phys. Sci.* **219**, 186–203 (1953).

10. Aris, R. On the dispersion of a solute in a fluid flowing through a tube. *Proc. R. Soc. London. Ser. A. Math. Phys. Sci.* **235**, 67–77 (1956).
